# Palmitoylethanolamide Ameliorates Hippocampal Damage and Behavioral Dysfunction After Perinatal Asphyxia in the Immature Rat Brain

**DOI:** 10.3389/fnins.2018.00145

**Published:** 2018-03-28

**Authors:** María I. Herrera, Lucas D. Udovin, Nicolás Toro-Urrego, Carlos F. Kusnier, Juan P. Luaces, Francisco Capani

**Affiliations:** ^1^Centro de Investigaciones en Psicología y Psicopedagogía, Facultad de Psicología, Universidad Católica Argentina, Buenos Aires, Argentina; ^2^Instituto de Investigaciones Cardiológicas, Universidad de Buenos Aires, Consejo Nacional de Investigaciones Científicas y Técnicas, Buenos Aires, Argentina; ^3^Facultad de Medicina, Universidad Católica Argentina, Buenos Aires, Argentina; ^4^Universidad Autónoma de Chile, Santiago de Chile, Chile

**Keywords:** perinatal asphyxia, palmitoylethanolamide, neuroprotection, hippocampus, CA1 region

## Abstract

Perinatal asphyxia (PA) is an obstetric complication associated with an impaired gas exchange. This health problem continues to be a determinant of neonatal mortality and neurodevelopmental disorders. Palmitoylethanolamide (PEA) has exerted neuroprotection in several models of brain injury and neurodegeneration. We aimed at evaluating the potential neuroprotective role of PEA in an experimental model, which induces PA in the immature rat brain. PA was induced by placing *Sprague Dawley* newborn rats in a water bath at 37°C for 19 min. Once their physiological conditions improved, they were given to surrogate mothers that had delivered normally within the last 24 h. The control group was represented by non-fostered vaginally delivered pups, mimicking the clinical situation. Treatment with PEA (10 mg/kg) was administered within the first hour of life. Modifications in the hippocampus were analyzed with conventional electron microscopy, immunohistochemistry (for NeuN, pNF-H/M, MAP-2, and GFAP) and western blot (for pNF H/M, MAP-2, and GFAP). Behavior was also studied throughout Open Field (OF) Test, Passive Avoidance (PA) Task and Elevated Plus Maze (EPM) Test. After 1 month of the PA insult, we observed neuronal nucleus degeneration in CA1 using electron microscopy. Immunohistochemistry revealed a significant increase in pNF-H/M and decrease in MAP-2 in CA1 reactive area. These changes were also observed when analyzing the level of expression of these markers by western blot. Vertical exploration impairments and anxiety-related behaviors were encountered in the OF and EPM tests. PEA treatment attenuated PA-induced hippocampal damage and its corresponding behavioral alterations. These results contribute to the elucidation of PEA neuroprotective role after PA and the future establishment of therapeutic strategies for the developing brain.

## Introduction

Perinatal asphyxia (PA) is an obstetric complication occurring when oxygen supply to the newborn is temporally interrupted (Adcock and Papile, 2008). This health problem remains as a condition of high prevalence (1 to 10/1000 live births) worldwide (Douglas-Escobar and Weiss, 2015). PA continues to be a determinant of neurological morbidity (Morales et al., 2011). If the region affected is immature, the insult interferes with the initial plastic changes necessary for the establishment of brain circuitries (Herrera-Marschitz et al., 2014). Several neurodevelopmental disorders (NDDs) have been associated with PA, including Attention Deficit Disorder (Perna and Cooper, 2012), Autism Spectrum Disorder (Schieve et al., 2014; Fezer et al., 2017) and distinct learning disorders (van Handel et al., 2007). Symptomatology becomes apparent during childhood, as environmental demands increase (Herrera-Marschitz et al., 2017).

An experimental model of PA was established several years ago (Bjelke et al., 1991). This rat model of global asphyxia involves the submersion of pup containing uterine horns in a water bath immediately after caesarean section, mimicking delayed labor. This method presents other translational advantages (Barkhuizen et al., 2017; Herrera et al., 2017; Herrera-Marschitz et al., 2017), such as the induction of PA at a time point when rodent brain maturity resembles the brain of a human fetus at 22–32 gestational weeks (Semple et al., 2013). This aspect is of clinical relevance since prematurity is associated with PA and increases the risk of neurological morbidity (Kuzniewicz et al., 2014). In the last 26 years, this murine model contributed to scientific advances on the neuropathological, functional (Barkhuizen et al., 2017) and synaptic effects of PA on the CNS (Herrera et al., 2017), and on mechanisms of neuroprotection (Herrera-Marschitz et al., 2011; Muñiz et al., 2014). Research on neuroprotection is based on the concept of secondary lesion, which offers a therapeutic window for early intervention (Blanco et al., 2011).

Palmitoylethanolamide (PEA) is an endogenous lipid compound derived from the reaction between ethanolamine and palmitic acid (Guida et al., 2017a). PEA is member of the N-acylethanolamides (NAEs) family, characterized for inhibiting mechanisms of secondary injury (Ahmad et al., 2012a). This pro-homeostatic mediator (Petrosino et al., 2010; Petrosino and Di Marzo, 2017) exerted neuroprotective effects when administered exogenously in models of brain injury and neurodegeneration (Herrera et al., 2016), including spinal cord injury (Genovese et al., 2008; Esposito et al., 2011), traumatic brain injury (Ahmad et al., 2012a; Guida et al., 2017a), stroke (Ahmad et al., 2012b), Alzheimer's Disease (D'Agostino et al., 2012; Scuderi et al., 2014), and Parkinson's Disease (Esposito et al., 2012). These effects are apparently mediated by the nuclear Peroxisome Proliferator-Activated Receptor (PPAR)-α (Lo Verme et al., 2005).

Recent studies from our laboratory reported a dysregulation of NAEs signaling system in rat hippocampus at post-natal day 30 (P30) after PA, and suggested PEA might exert neuroprotective effects when administered exogenously (Blanco et al., 2015). P30 constitutes an interesting time point for testing the effect of neuroprotective agents since it is equivalent to 4–11 human years (Semple et al., 2013), a period of NDDs onset (Herrera-Marschitz et al., 2014, 2017). In addition, the hippocampus represents a brain region of major vulnerability to PA (Petito and Pulsinelli, 1984; Pulsinelli, 1985; Van de Berg et al., 2000) and of considerable implication in the pathophysiology of NDDs (Rojas et al., 2004; Saraceno et al., 2016). Therefore, the aim of the present work was to study the neuroprotective role of PEA in rat hippocampus at P30 after PA, contributing to the establishment of treatments for PA and the consequent prevention of NDDs.

## Materials and methods

All procedures were approved by the Institutional Animal Care and Use Committee at the University of Buenos Aires (CICUAL#4091/04) and conducted according to the principles of the Guide for the Care and Use of Laboratory Animals (Animal Welfare Assurance, A-3033-01/protocol#S01084).

### Animals

Subjects consisted of 20 pregnant *Sprague Dawley* rats obtained from the central vivarium at the School of Veterinary Sciences, Universidad de Buenos Aires. Animals arrived 1 week prior to delivery to our local vivarium in order to acclimate to the new environment. They were housed in individual cages with constant temperature (21 ± 2°C) and humidity (65 ± 5%) conditions. A light/dark cycle of 12:12 h was employed. The light period began at 7 a.m. Food and tap water were provided.

### Induction of PA

Rat pups were subjected to PA using the experimental model originally developed by Bjelke et al. (1991) and modified in our laboratory (Capani et al., 2009). At the expected day of delivery, gestational day 22, pregnant rats were observed and when the first pup was delivered, the dam was immediately euthanized by decapitation. Uterine horns were rapidly isolated through an abdominal incision, and submerged in a water bath at 37°C for 19 min, inducing severe asphyxia. When time elapsed, horns were rapidly opened and pups were subjected to tactile intermittent stimulation until regular breathing was established. Umbilical cord was tied and the animals were left to recover 1 h under a heat lamp.

### Protocol of neuroprotection

Sixty-three male rat pups were treated with PEA or vehicle (VHI) in a dose of 10 mg/kg by subcutaneous injection. This dose has shown effective results in several models of brain injury and neurodegeneration (Genovese et al., 2008; Esposito et al., 2011, 2012; Ahmad et al., 2012a,b; Sayd et al., 2014; Scuderi et al., 2014). The respective treatment was performed within the first hour of life. VHI used was 1:1:8 DMSO, Tween 80 and NaCl. The formulation of PEA tested was C18H37NO2 from Tocris Bioscience (Bristol, UK). This formulation crosses the blood-brain barrier because it is proven that this compound possess neuromodulatory effects *in vivo* through inhibition of nicotine-induced excitation on dopamine neurons in the ventral tegmental area of rats (Melis et al., 2008). Besides, this compound exhibits antinociceptive, antiepileptic and anticonvulsant properties *in vivo* (Lambert et al., 2001; Sheerin et al., 2004). On the other hand, it is widely accepted that this PEA formulation is involved in the modulation of neuroinflammation produced in the central nervous system (Sayd et al., 2014). It has shown to be useful in the control of neuropathic pain (Re et al., 2007) and it could exert neuroprotective properties in neurological disorders such as stroke (Ahmad et al., 2012b), traumatic brain injury (Ahmad et al., 2012a), Alzheimer and Parkinson's disease (D'Agostino et al., 2012; Esposito et al., 2012) or addiction (Coppola and Mondola, 2013).

Once pups improved their physiological conditions, they were given to surrogate mothers that had delivered normally within the last 24 h. Four experimental groups were established: rats subjected to PA and injected with VHI (PA group, *n* = 15), rats born vaginally and injected with VHI (control – CTL- group, *n* = 13), rats subjected to PA and injected with PEA (PA+PEA group, *n* = 18) and rats born vaginally and injected with PEA (CTL+PEA group, *n* = 17). The different groups were marked and mixed in litters with a surrogate mother. After weaning, rats were housed in cages of 3–4 animals from the same group. Cesarean controls were not used since previous studies revealed this group did not present significant differences with vaginal controls (Galeano et al., 2011; Blanco et al., 2015).

### Behavioral testing

#### General procedure

At P30, rats (*N* = 63) were subjected to behavioral tests in an isolated behavioral room from 8 a.m. to 5 p.m. In order to avoid the confounding effect of time of day, testing order of the groups was counterbalanced. The apparatus of each test was cleaned with 70 % ethanol between sessions to minimize the olfactory stimulus. White noise was provided during tests (Galeano et al., 2015). Before any of the tests, the assessed rat was placed in the behavioral room for acclimatization during 5 min (Molina et al., 2016). In this way, potential confounding variables were controlled. The following tests were performed.

#### Elevated plus maze (EPM) test

The EPM test evaluates anxiety-related behaviors, which are hippocampal dependent (Montgomery, 1955; Brenes et al., 2009; Violle et al., 2009; Molina et al., 2016). The apparatus was made of black melamine and consisted of a central square platform (11 × 11 cm). Four illuminated arms radiated from this platform: two closed arms (50 × 11 × 40 cm) and two open arms (50 × 11 × 0.25 cm), conforming a maze in the shape of a plus. This maze was elevated to a height of 100 cm above floor-level (Galeano et al., 2015). Procedure: The test began by placing the rat on the central platform facing an open arm. The rat was allowed to freely explore the apparatus for 5 min. When time elapsed, the rat was removed from the apparatus and returned to the home cage. A rat was considered to enter an arm when its 4 paws were inside (Galeano et al., 2015). Total distance traveled and time spent in closed arms were quantified as indicators of locomotion. Time spent in open arms was measured in order to assess anxiety levels. The stretching of rats over the edge of an open arm, known as head-dipping (HD), was also recorded as a risk-assessment-behavior (RAB) (Rodgers and Cole, 1993; Molina et al., 2016). Prototypical behaviors, rearing and grooming, were assessed. Time spent rearing was recorded as a hippocampal-dependent indicator of vertical exploration (Lever et al., 2006). Rats that fell down from the maze during testing were excluded from the study. Testing sessions were filmed using a digital camera (Sony HDR-AS100V) and later analyzed with ANY-Maze video-tracking software (version 5.29). Rearing, grooming and HD were registered by blind evaluation and time was quantified using the key resource of ANY-Maze.

#### Open field (OF) test

The OF test was used to assess locomotion, exploratory activity and changes in emotionality induced by exposure to a novel environment (Molina et al., 2016). These behavioral parameters also depend on hippocampal integrity (Vianna et al., 2000; Barros et al., 2006; Molina et al., 2016). The apparatus consisted of a black melamine square (60 × 60 × 40 cm) surrounded by walls of 40 cm high (Galeano et al., 2015). Procedure: Rats were placed on the center of the apparatus and allowed to explore it for 5 min. (Molina et al., 2016). Total distance traveled and number of line crossings were quantified in order to measure locomotion. Prototypical behaviors, rearing and grooming, were assessed. Time spent rearing was recorded as a hippocampal-dependent indicator of vertical exploration (Lever et al., 2006). Testing sessions were filmed using a digital camera (Sony HDR-AS100V) and later analyzed with ANY-maze video-tracking software (version 5.29). Rearing and grooming were registered by blind evaluation and time was quantified using the key resource of ANY-maze.

#### Inhibitory avoidance (IA) task

The IA task measures memory of an aversive experience through the avoidance of a location where an unpleasant experience occurred (Molina et al., 2016; Saraceno et al., 2016). This parameter constitutes an indicator of associative memory, which is hippocampal-dependent (Ennaceur and Delacour, 1988; Lorenzini et al., 1996; Izquierdo and Medina, 1997; Molina et al., 2016). The apparatus consisted of a black melamine square (60 × 60 × 40 cm) divided into two compartments: one was illuminated and the other was dark. A removable cover was used for this purpose. The floor of the dark compartment was made of a stainless steel grid, which could deliver an electric shock when pressing a button. Procedure: The test was divided in three sessions. The habituation session consisted of placing the rat in the illuminated compartment. After entering the dark section three times, the rat was removed from the apparatus. If the rat did not enter the dark side three times, it was removed after the three-minute trial was completed. The next trial was performed after 10 min by placing the rat again in the lit compartment. When it entered the dark section, doors were closed retaining the rat for 10 s inside this compartment. The training session took place after an hour. The rat was placed in the illuminated section and the latency to enter the dark side was registered. As soon as the rat entered the dark compartment, an electric shock (1.2 mA, 2s) was delivered. Then, the rat was returned to its home cage. One hour later, the rat was placed in the lit compartment but this time the electric shock was not delivered. This is the retention session, when the latency to enter the dark side is again measured. The ratio between the latency to enter the dark compartment in the retention (T2) and the training session (T1), which is expressed as T2/T1, constitutes an indicator of aversive associative memory (Molina et al., 2016).

#### Order of testing

Tests were performed in the order they were described above. As the EPM test is specific for measuring anxiety, it was performed firstly, in order to avoid the confounding effect of previous tests. Inversely, the IA task was administered at the end because the aversive effect of the electric shock could constitute a confounding variable for following tests.

### Tissue fixation and immunohistochemistry

At P30, intracardiac perfusion and coronal hippocampal sections were performed as described previously (Saraceno et al., 2016). Four animals per treatment (four replicates) were used. Three sections were immunostained for each analyzed brain. Animals were anesthetized with intraperitoneal administration of ketamine 40 mg/kg and xylazine 5 mg/kg, and perfused intracardiacally with 4% paraformaldehyde in 0.1 M phosphate buffer, pH 7.4. Brains were removed and post-fixed in the same fixative solution for 2 h at room temperature, and then immersed overnight at 4°C in 0.1 M phosphate buffer, pH 7.4. Coronal hippocampal sections (50 μm thickness) were obtained using a Vibratome (VT 1000 S, Leyca Microsystems, Wetzlar, Germany). Immunohistochemistry was performed on free-floating sections under moderate shaking. Endogenous peroxidase was quenched (3% H_2_O_2_, 70% methanol, 30% H_2_O_2_ 30 vol) and non-specific labeling was blocked using 0.3% normal goat serum. Free-floating sections were incubated overnight at 4°C with anti-neuron-specific nuclear protein (NeuN; 1:1,000, mouse-IgG; Millipore), anti-microtubule-associated protein 2 (MAP-2; 1:250, polyclonal rabbit-IgG; Abcam), anti-phosphorylated high and medium molecular weight neurofilaments (pNF H/M; 1:500, monoclonal mouse-IgG; Millipore) or anti-glial fibrillary acidic protein (GFAP; monoclonal rabbit IgG, 1:200, Cell Marque, a Sigma-Aldrich Company). Then sections were incubated for 2 h at room temperature (RT) with secondary antibodies (Biotinylated anti-mouse-IgG, 1:500, Vector; Biotinylated anti-rabbit-IgG, 1:500, Vector). Amplification was done using avidin-biotinylated horseradish peroxidase complex (1:500, Dako) in PBS for 1 h, followed by washing in PBS before chromogen development (DAB; Cell Marque, a Sigma-Aldrich Company). Light microscopic images were obtained (using a Leyca microscopy).

### Electron microscopy procedure

Coronal brain sections of four animals were cut at a thickness of 40 μm with a vibratome through the level of the dorsal *neostriatum* and post-fixed for 1 h with 4% paraformaldehyde in 0.1 M cacodylate buffer, pH 7.4. Then, tissue sections from hypoxic and control animals were stained by conventional osmium–uranium–lead method, following the protocol described in Capani et al. (2001). Thin sections (50-80 nm) were cut with Reichert Ultracut E by using glass knives. Sections were counterstained with a combination of lead citrate. Thin sections were examined using a JEOL 200CX electron microscope at 80–100 keV.

### Morphometric analysis

One hundred fifty by 150 μm was sampled in each photo in order to estimate the percentage of reactive area for pNF-H/M and MAP-2 using Image J Program (Image J 1.41o, NIH, USA). We focused our analysis on CA1 area of hippocampus since it is one of the most affected areas by PA insult (Petito and Pulsinelli, 1984; Pulsinelli, 1985; Van de Berg et al., 2000). The number of GFAP immunoreactive astrocytes was estimated manually in the *stratum radiatum* of CA1 hippocampal area. A total of 80 counting frames was assessed per animal. A blind observer selected 5 fields for each sector from 4 sections of *striatum* and 10 fields of cortex from 10 sections (two hundred fields of *striatum* and 200 fields of cortex). To estimate the percentage of reactive area the experiments were repeated at least 3 times.

### Western blot

Western blot analysis was performed using four animals per treatment (four replicates). Experiments were repeated three times for each brain. Animals were euthanized by decapitation, brains were dissected, homogenized in ice-cold lysis buffer (10 mM Tris/HCl, pH 7.4, 10 mM NaCl, 3 mM MgCl2, 0.1% Triton X-100, protease inhibitors). Tissues were thawed on ice and centrifuged at 14,000 rpm for 15 min at 4°C. The supernatants were analyzed for total protein concentration using Bradford solution (Bio-Rad, Richmond CA, USA) in 96-well plates using bovine serum albumin (BSA) as standard. 90 μg of total protein were diluted in sample buffer (0.3 M Tris/HCl, pH 7, 50% glycerol, 5%SDS, 1 mM EDTA, 0.1% bromophenol blue). The samples were subjected to SDS-PAGE using the Mini-protein II cell (Bio-Rad, Richmond CA, USA) with precast 4–20% Precise gels (Bio-Rad, Richmond CA, USA). Proteins were transferred to PVDF membranes (MACHEREY-NAGEL, Germany) using the semi-dry transfer unit Hoefer TE 70 (Amersham Biosciences). Membranes were blocked with 5% non-fat milk powder and 1% BSA in Tris-buffered saline containing 0.05% Tween 20 and incubated overnight at 4°C with anti-microtubule-associated protein 2 (MAP-2; 1:1,000, polyclonal rabbit-IgG; Abcam), anti-phosphorylated high and medium molecular weight neurofilaments (pNF-H/M; 1:500, monoclonal mouse-IgG; Millipore) and anti-glial fibrillary acidic protein (GFAP; monoclonal mouse-IgG, 1:1,000; Santa Cruz Biotechnology). We used anti glyceraldehyde-3-phosphate dehydrogenase (GAPDH, 1:1,000, rabbit-IgG, Sigma-Aldrich) as loading control. Blots were rinsed three times in PBS with 0.5% Tween-20 buffer (PBST), and then incubated with the corresponding horseradish peroxidase (HRP)-conjugated secondary antibody (1:3,000,Bio-Rad, Richmond CA, USA) for 1 h at RT. Immunoreactive bands were detected using an ECL western blotting analysis system (clarity western ECL substrate, Bio-Rad). Films were scanned and the optical density of protein bands was quantified using Gel Pro Analyzer 3.1.00.00 (Media Cybernetics).

### Statistical analysis

Results were expressed as means ± SEM. Normal distribution and equality of variances were checked using Shapiro-Wilk test and Levene's test, respectively. Statistical analyses were conducted by two-way analysis of variances (ANOVAs) with birth condition (CTL and PA) and treatment (VHI and PEA) as the main factors. When interaction effects were significant, analyses were performed by *post hoc* comparisons using Student's *t*-test (two-tailed) adjusted by Bonferroni correction. Differences with a probability of 5% or less were considered to be significant. Statistical analysis was carried out using Graph pad Prism 5 program.

## Results

### Vulnerability of CA1 hippocampal neurons to PA. effects of PEA treatment

**Figure 1:**
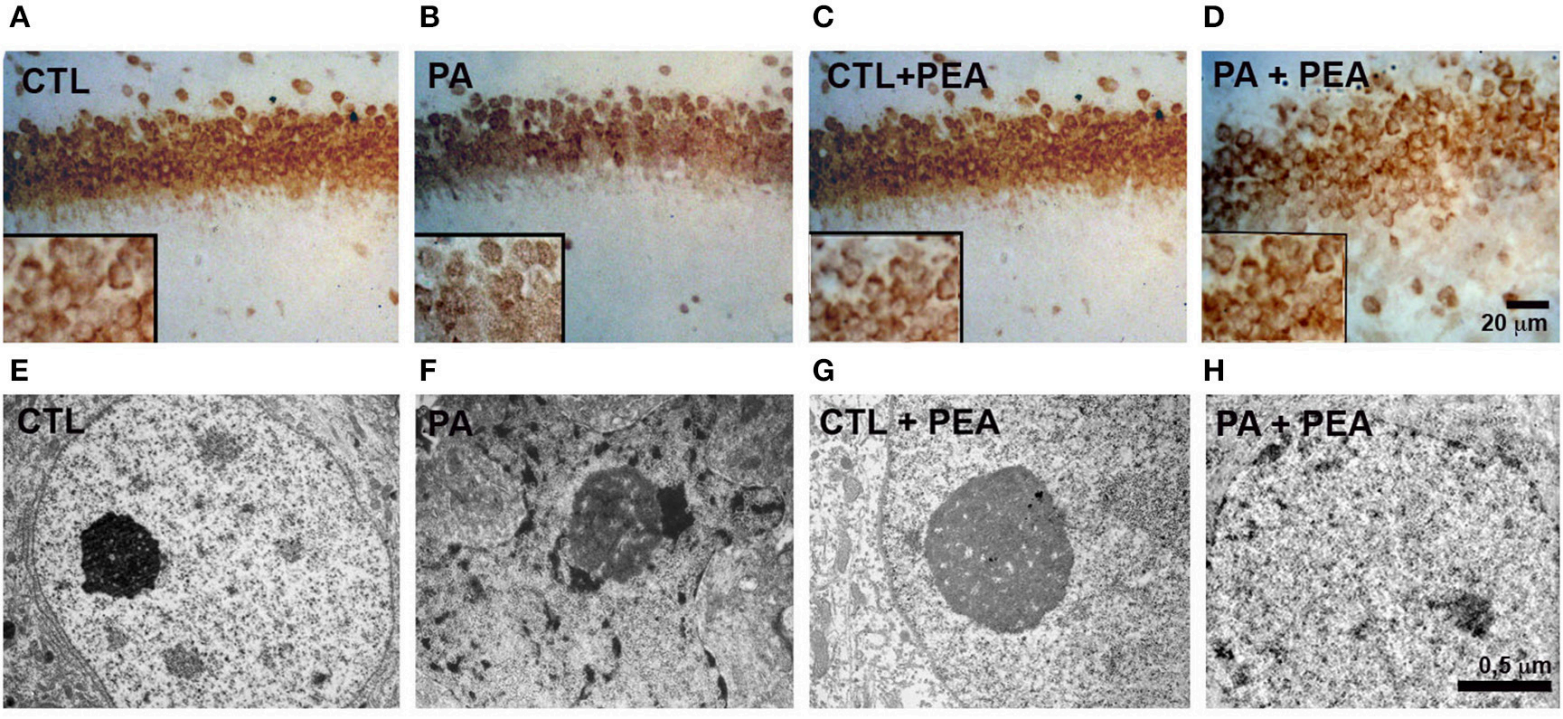
Neuronal alterations induced by PA and recovery after PEA treatment. (**A-D)** Micrographs of Stratum radiatum of CA1 hippocampal area in the different groups analyzed by NeuN Immunostaining. (**E–H)**. Electron microscopy (EM) of CA1 neurons showing that most of the condensed cells correspond to neurons in degeneration. Statistical analysis of different parameters was performed. Significant differences were calculated by two-way analysis of variances (ANOVAs) tests followed by *post hoc* comparisons using Student's *t*-test (two-tailed) adjusted by Bonferroni correction.

Nuclear morphology was analyzed to evaluate the vulnerability of CA1 neurons at the *stratum radiatum* of rat hippocampus at P30 (Figure 1; Table 1). We observed a significant increase (*F* = 2142, *p* < 0.05) in the number of pyknotic nucleus in the hippocampal CA1 neurons layer of asphyctic animals (Table 1). In addition, the number of pyknotic nucleus was significantly reduced in the PA+PEA group (*F* = 1978, *p* < 0.05) (Table 1). Then we analyzed NeuN+ neurons and classified them in two categories: (a) normal neurons, characterized by an intense NeuN+ nucleus and (b) abnormal neurons, with NeuN fragmentation of the nucleus, or cytoplasmic staining without nuclear staining (Robertson et al., 2006). We observed a significant increase in the number of abnormal neurons in the hippocampal CA1 layer of asphyctic animals (*F* = 2036, *p* < 0.05) (Table 1; Figures 1A,B). PEA treatment improved significantly nucleus alterations (*F* = 1936, *p* < 0.05) (Table 1; Figures 1B,D). Electron microscopy analyses showed clear degeneration of CA1 neurons after PA (Figures 1E,F), which was reduced after treatment with PEA (Figures 1F–H). Altogether, these results indicate that major PA effects occurred on CA1 neurons and could be ameliorated by PEA treatment.

**Table 1:** Damage of CA1 hippocampal neurons after PA and effect of PEA treatment.

**Groups**	**Pyknotic nuclei**	**NeuN + neurons**	**Normal neurons**	**Abnormal neurons**
CTL	13.24 ± 1.42	76.35 ± 1.56	71.24 ± 0.33	5.11 ± 0.51
PA	20.96 ± 0.21^*^	64.24 ± 3.54	53.44 ± 0.82	10.8 ± 0.71^*^
CTL+PEA	12.34 ± 1.33	73.23 ± 2.32	69.25 ± 0.93	5.98 ± 0.43
PA+PEA	15.36 ± 1.97	67.26 ± 2.48	63.35 ± 0.72	7.91 ± 0.64

*Data expressed as mean ± SD. Statistical differences were assessed by two-way ANOVA*.

* *P < 0.05*.

### PA-induced alterations in pNF-H/M. protective effect of PEA treatment

**Figure 2:**
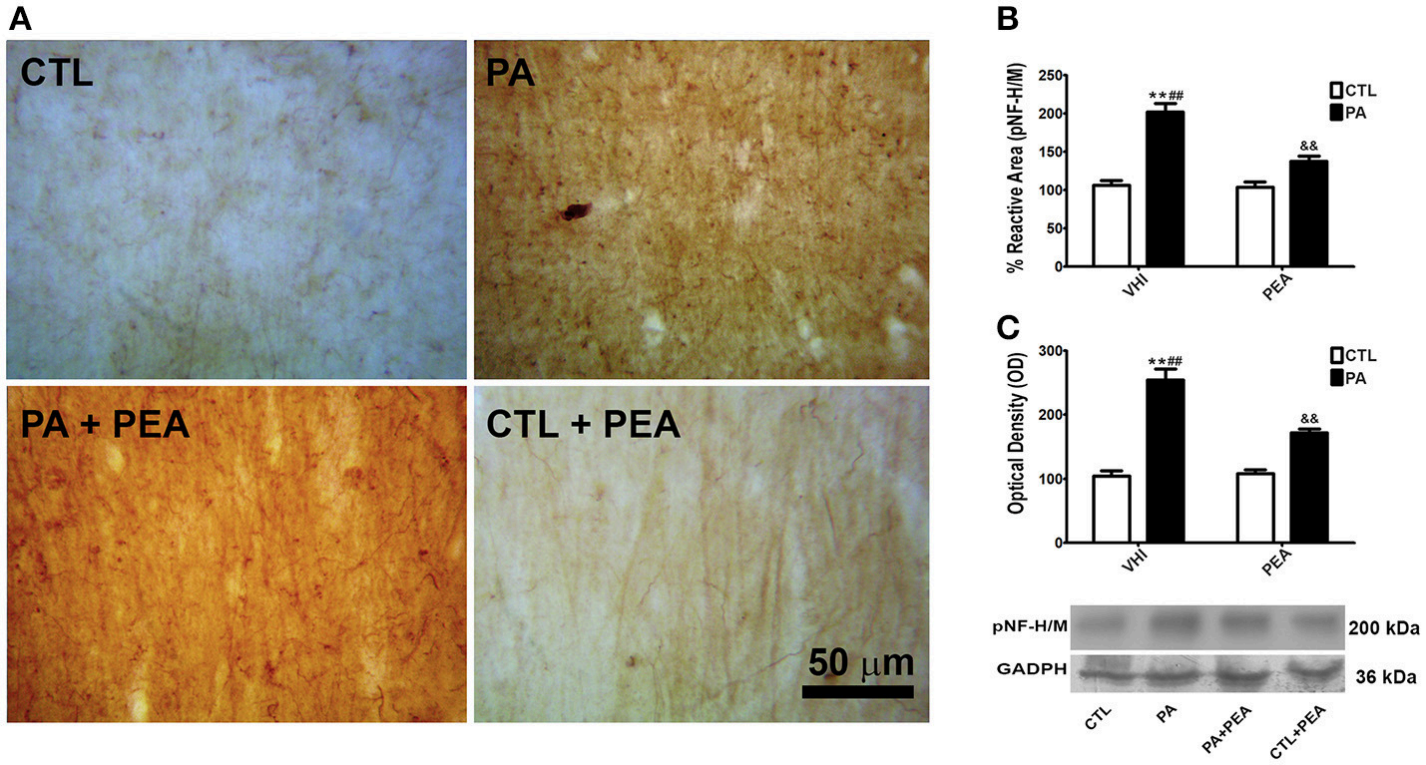
pNF-H/M immunostaining and protein expression levels in the rat hippocampus. PEA treatment attenuated increased pNF-H/M expression after PA. **(A)** Representative image of the striatum radiatum of CA1 hippocampal area immunostained for pNF-H/M in the different groups. **(B)** The statistical analysis revealed a significant increase in the percentage of reactive area of pNF-H/M inmunostaining in PA rats treated with vehicle (PA) in comparison to CTL rats treated with vehicle (CTL), and a significant decrease in the percentage of reactive area of pNF-H/M inmunostaining in PA rats treated with PEA (PA+PEA) in comparison to PA rats treated with vehicule (PA), suggesting a partial reversion of axonal dysfunction and degeneration associated with perinatal asphyxia, although PA+PEA group did not reach values similar to controls. PEA by itself did not produce any differences in the percentage of reactive area of pNF-H/M inmunostaining, since CTL+PEA rats did not differ from CTL rats. **(C)** Consistent with these morphological observations, results similar to those of the percentage of reactive area were observed by western blot in protein expression levels of pNF-H/M. Bars and error bars represent mean + SEM calculated by two-way analysis of variances (ANOVAs) tests followed by *post hoc* comparisons using Student's *t*-test (two-tailed) adjusted by Bonferroni correction. ^**^P < 0.001, PA vs. CTL; ^*##*^*P* < 0.001, PA vs. PA+PEA; ^&&^*P* < 0.001, PA+PEA vs. CTL.CTL, Control group; PA, rats subjected to PA; PA+PEA, rats subjected to PA and PEA treatment; CTL+PEA, control group subjected to PEA treatment.

The accumulation of pNF-H/M as a measure of axonal dysfunction and degeneration was analyzed by immunostaining. Figure 2A shows a representative example of the *stratum radiatum* of CA1 hippocampal section immunostained for pNF-H/M. The two-way ANOVA revealed that the main factors of birth condition and treatment were both significant when analyzing the percentage of reactive area for pNF-H/M (*F* = 2033, *p* < 0.0001; *F* = 509.8, *p* < 0.0001, respectively), and the interaction was also significant (*F* = 604.8, *p* < 0.0001). *Post hoc* analysis of the simple effects indicated that the reactive area for pNF-H/M at P30 showed a significant increase as a consequence of PA (*t* = 49.27, *p* < 0.001) (Figure 2B). This alteration was significantly attenuated in PA+PEA group (*t* = 31.54, *p* < 0.001), although this group did not reach values similar to controls (*t* = 14.49, *p* < 0.001) (Figure 2B). Consistent with these morphological observations, birth condition and treatment were significant with respect to protein expression for pNF-H/M (*F* = 2494, *p* < 0.0001; *F* = 517.9, *p* < 0.0001, respectively). The interaction was also significant (*F* = 477.1, *p* < 0.0001). An increase in pNF-H/M expression was observed in PA group (*t* = 50.76, *p* < 0.001), and reduced after PEA treatment (*t* = 13.66, *p* < 0.001) (Figure 2C). However, PA+PEA group still presented significant differences with controls (*t* = 19.87, *p* < 0.001) (Figure 2C). Treatment with PEA in CTL rats had no significant effect on both reactive area and protein expression for pNF-H/M in comparison to CTL rats injected with VHI (*t* = 0.6472, *p* > 0.05; *t* = 0.09113, *p* > 0.05, respectively) (Figures 2B,C, respectively).

### Dendritic cytoskeleton alteration and its attenuation by PEA treatment

**Figure 3:**
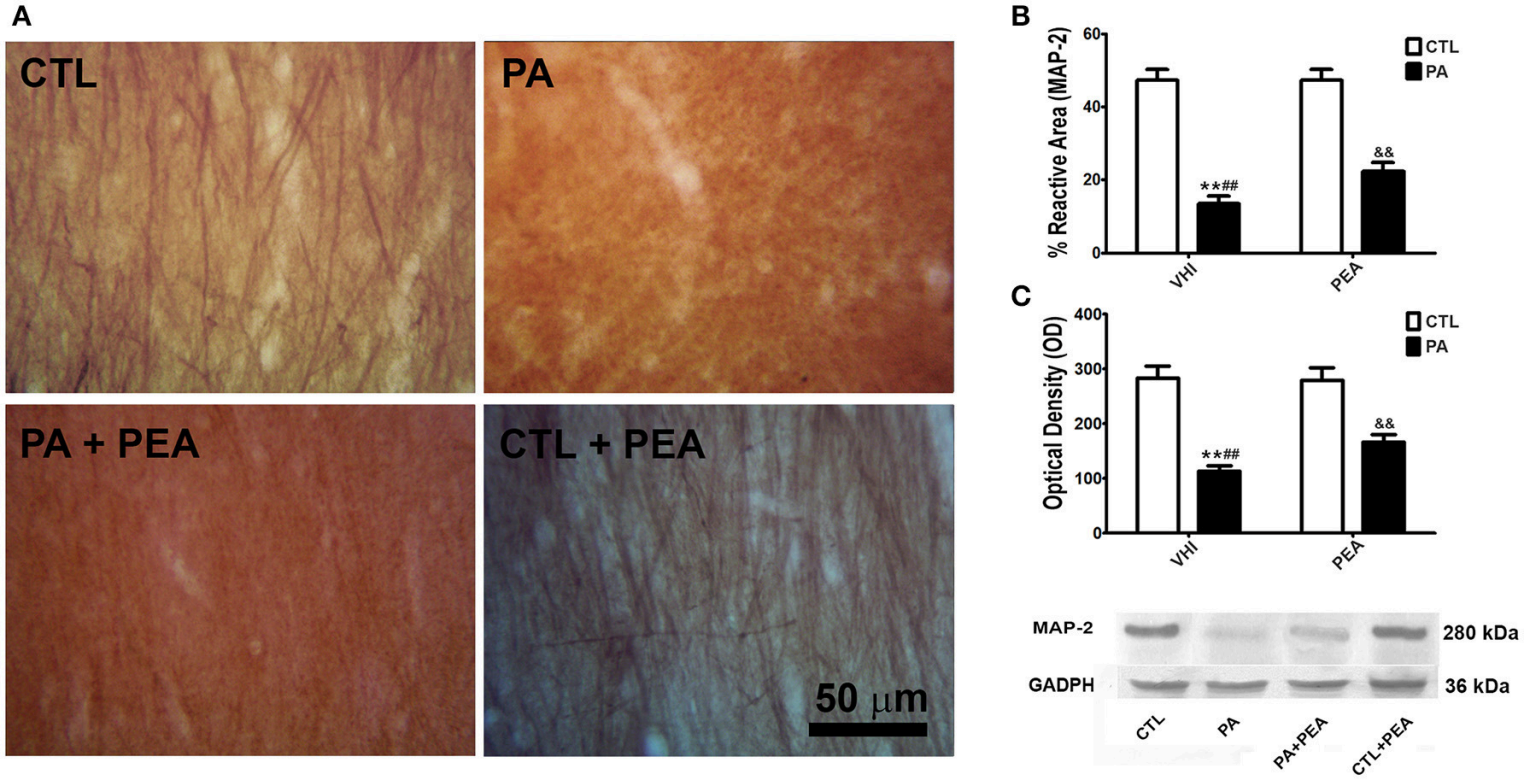
MAP-2 immunostaining and protein expression levels in the rat hippocampus. **(A)** Representative image of the striatum radiatum of CA1 hippocampal area immunostained for MAP-2 in the different groups. **(B)** The statistical analysis revealed a significant decrease in the percentage of reactive area of MAP-2 positive dendrites in PA rats treated with vehicle (PA) in comparison to CTL rats treated with vehicle (CTL), and a significant increase in the percentage of reactive area of MAP-2 positive dendrites in PA rats treated with PEA (PA+PEA) in comparison to PA rats treated with vehicule (PA), suggesting a partial reversion of dendritic alterations associated with perinatal asphyxia, although PA+PEA group did not reach values similar to controls. PEA by itself did not produce any differences in the percentage of reactive area of MAP-2 positive dendrites, since CTL+PEA rats did not differ from CTL rats. **(C)** Consistent with these morphological observations, results similar to those of the percentage of reactive area were observed by western blot in protein expression levels of MAP-2. Bars and error bars represent mean + SEM calculated by two-way analysis of variances (ANOVAs) tests followed by *post hoc* comparisons using Student's *t*-test (two-tailed) adjusted by Bonferroni correction. ^**^P < 0.001, PA vs. CTL; ^*##*^*P* < 0.001, PA vs. PA+PEA; ^&&^*P* < 0.001, PA+PEA vs. CTL.CTL, Control group; PA, rats subjected to PA; PA+PEA, rats subjected to PA and PEA treatment; CTL+PEA, control group subjected to PEA treatment.

Since PA affected neuronal population, we also studied cytoskeleton organization of neural processes. Changes in dendrite morphology were analyzed through immunostaining of a dendrite-specific marker, MAP-2 (Figure 3A). According to two-way ANOVA results for MAP-2 reactive area, both birth condition and treatment were significant (*F* = 1342, *p* < 0.0001; *F* = 72.89, *p* < 0.0001, respectively). The interaction was also significant (*F* = 59.01, *p* < 0.0001). *Post hoc* analysis revealed the PA group presented a significant decrease in the percentage of MAP-2 reactive area in comparison to the CTL group (*t* = 31.33, *p* < 0.001) (Figure 3B). This reduction in MAP-2 reactive area was partially reversed after PEA treatment (*t* = 11.47, *p* < 0.001), without reaching values similar to controls (*t* = 20.47, *p* < 0.001) (Figure 3B). These morphological findings were confirmed by western blot. Birth condition and treatment were significant factors for MAP-2 protein expression (*F* = 5181, *p* < 0.0001; *F* = 316.7, *p* < 0.0001, respectively), and the interaction was significant (*F* = 289.7, *p* < 0.0001). A significant reduction in MAP-2 expression was observed as a consequence of PA (*t* = 62.93, *p* < 0.001) (Figure 3C). This modification was attenuated in the PA+PEA group (*t* = 24.62, *p* < 0.001). However, this group still presented significant differences with controls (*t* = 38.86, *p* < 0.001) (Figure 3C). Finally, treatment with PEA did not exert a significant effect either in MAP-2 reactive area or in protein expression when comparing CTL and CTL+PEA groups (*t* = 0.6053, *p* > 0.05; *t* = 0.5479, *p* > 0.05, respectively) (Figures 3B,C, respectively).

### GFAP immunostaining and protein expression at P30 after PA

We used immunohistochemistry and western blot to analyze glial response to PA injury and PEA treatment. As regards the number of GFAP positive astrocytes, the two-way ANOVA indicated that birth condition and treatment were not significant (*F* = 0.0009796, *p* = 0.9752; *F* = 0.02149, *p* = 0.884). These results were confirmed when analyzing the expression of GFAP. Birth condition and treatment were not significant either (*F* = 1.194, *p* = 0.2853; *F* = 0.9972, *p* = 0.328, respectively).

### Behavioral modifications induced by PA at P30. protective role of PEA

Locomotion and general activity were not affected by PA at P30. With respect to the number of line crossings in the OF test, the two-way ANOVA revealed neither birth condition (*F* = 1.93, *p* = 0.1716), nor treatment (*F* = 0.6706, *p* = 0.4172) were significant. Similarly, birth condition and treatment were not significant for time spent in closed arms in the EPM test (*F* = 0.03662, *p* = 0.8493; *F* = 3.624, *p* = 0.0646, respectively). In addition, birth condition and treatment were not significant for total distance traveled in the OF (*F* = 0.8553, *p* = 0.3592; *F* = 0.005146, *p* = 0.9431, respectively) and EPM test (*F* = 0.1234, *p* = 0.7272; *F* = 2.645, *p* = 0.1119, respectively).

**Figure 4:**
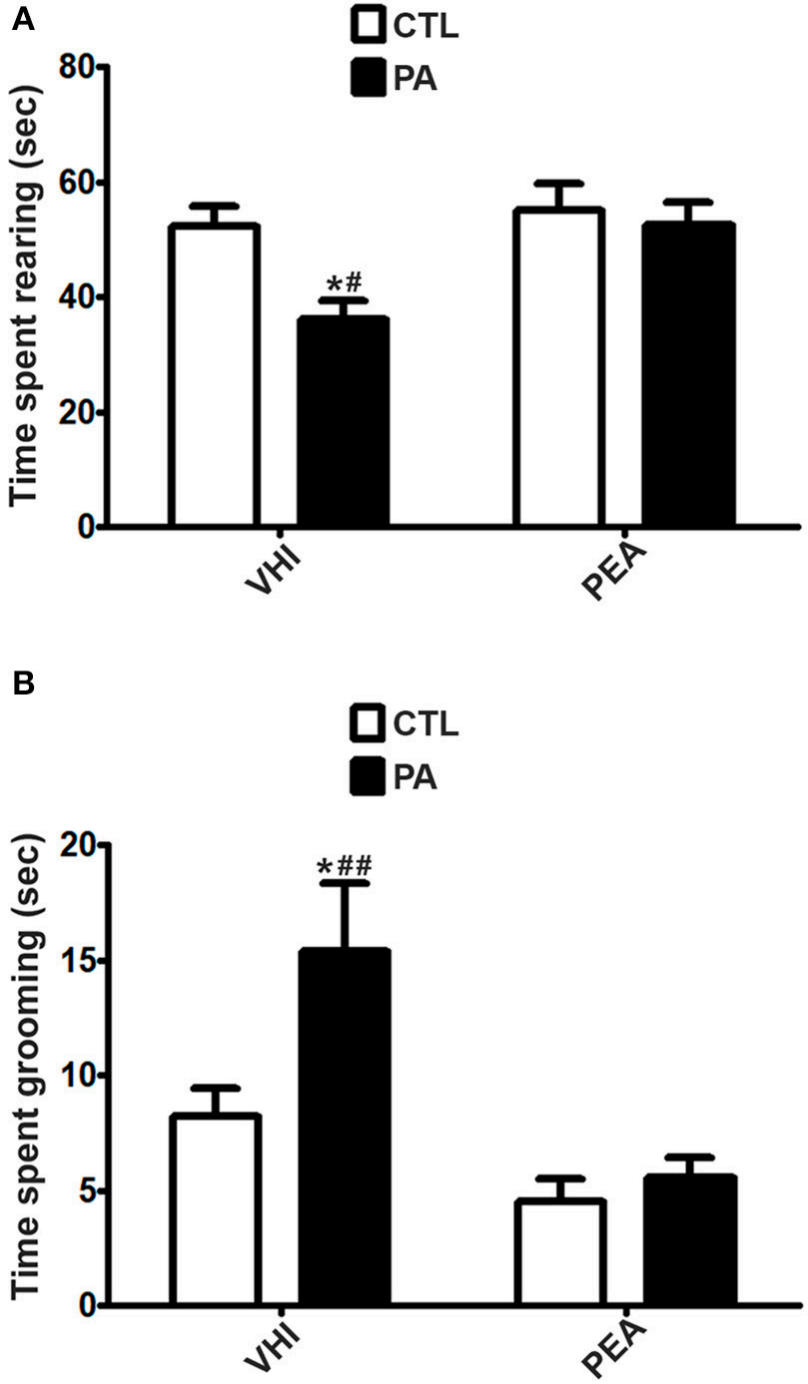
Behavioral differences in time spent rearing and time spent grooming between the 4 groups in the OF test. **(A)**. The statistical analysis revealed a significant reduction in time spent rearing in PA rats treated with vehicle (PA) in comparison to CTL rats treated with vehicle (CTL) and PA rats treated with PEA (PA+PEA). In addition, PA+PEA rats did not show significant differences on time spent rearing in comparison to CTL rats, suggesting a reversion of this behavioral alteration associated with perinatal asphyxia. PEA by itself had no significant effect on time spent rearing, since CTL+PEA rats did not differ in time spent rearing from CTL rats. **(B)** The statistical analysis indicated time spent grooming was significantly augmented in PA rats treated with vehicle (PA) in comparison to CTL rats treated with vehicle (CTL) and PA rats treated with PEA (PA+PEA). In addition, PA+PEA rats did not show significant differences on time spent grooming in comparison to CTL rats, suggesting a reversion of this behavioral alteration associated with perinatal asphyxia. PEA by itself had no significant effect on time spent grooming, since CTL+PEA rats did not differ in time spent grooming from CTL rats. Bars and error bars represent mean + SEM calculated by two-way analysis of variances (ANOVAs) tests followed by *post hoc* comparisons using Student's *t*-test (two-tailed) adjusted by Bonferroni correction. ^*^P < 0.05, PA vs. CTL; ^#^*P* < 0.01 and ^*##*^*P* < 0.001, PA vs. PA+PEA.CTL, Control group; PA, rats subjected to PA; PA+PEA, rats subjected to PA and PEA treatment; CTL+PEA, control group subjected to PEA treatment.

**Figure 5:**
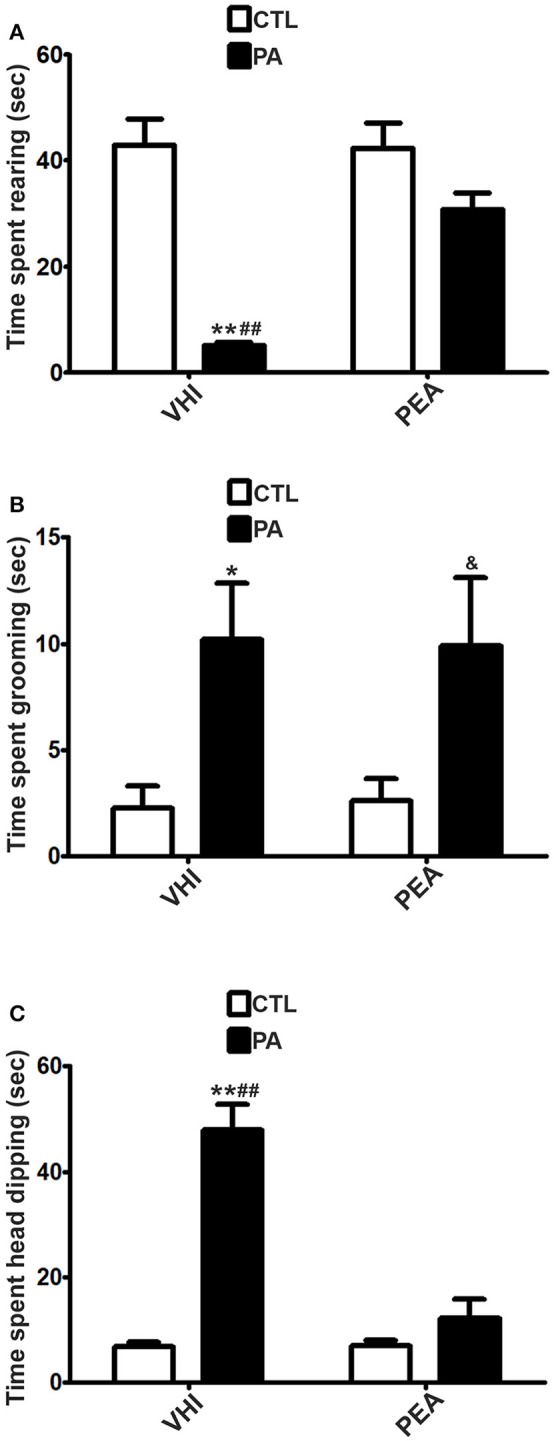
Behavioral differences in time spent rearing, time spent grooming and time spent head dipping between the 4 groups in the EPM test. **(A)** The statistical analysis revealed a significant reduction in time spent rearing in PA rats treated with vehicle (PA) in comparison to CTL rats treated with vehicle (CTL) and PA rats treated with PEA (PA+PEA). In addition, PA+PEA rats did not show significant differences on time spent rearing in comparison to CTL rats, suggesting a reversion of this behavioral alteration associated with perinatal asphyxia. PEA by itself had no significant effect on time spent rearing, since CTL+PEA rats did not differ in time spent rearing from CTL rats. **(B)** The statistical analysis indicated time spent grooming was significantly augmented in PA rats treated with vehicle (PA) in comparison to CTL rats treated with vehicle (CTL), but PA rats treated with PEA (PA+PEA) did not differ on time spent grooming in comparison to PA rats treated with vehicule (PA), suggesting that PEA did not exert a protective effect in PA rats. In addition, PA+PEA rats presented significant differences on time spent grooming in comparison to CTL rats. PEA by itself had no significant effect on time spent grooming, since CTL+PEA rats did not differ in time spent grooming from CTL rats. **(C)** The statistical analysis indicated time spent head dipping was significantly augmented in PA rats treated with vehicle (PA) in comparison to CTL rats treated with vehicle (CTL) and PA rats treated with PEA (PA+PEA). In addition, PA+PEA rats did not show significant differences on time spent head dipping in comparison to CTL rats, suggesting a reversion of this behavioral alteration associated with perinatal asphyxia. PEA by itself had no significant effect on time spent head dipping, since CTL+PEA rats did not differ in time spent head dipping from CTL rats. Bars and error bars represent mean + SEM calculated by two-way analysis of variances (ANOVAs) tests followed by *post hoc* comparisons using Student's *t*-test (two-tailed) adjusted by Bonferroni correction. ^*^P < 0.05 and ^**^P < 0.001, PA vs. CTL; ^*##*^*P* < 0.001, PA vs. PA+PEA; ^&^*P* < 0.05, PA+PEA vs. CTL. CTL, Control group; PA, rats subjected to PA; PA+PEA, rats subjected to PA and PEA treatment; CTL+PEA, control group subjected to PEA treatment.

In contraposition, vertical exploration might be altered at P30 as a consequence of PA, as it can be inferred from results in rearing, a prototypical behavior in rats. As for time spent rearing in the OF test, the two-way ANOVA revealed that the main factors of birth condition and treatment were significant (*F* = 5.51, *p* = 0.0226; *F* = 5.779, *p* = 0.0197, respectively). However, the interaction was not significant (*F* = 2.888, *p* = 0.095). *Post hoc* analysis indicated PA rats showed a significant decrease in time spent rearing with respect to the CTL group (*t* = 2.838, *p* < 0.05). This behavioral alteration was reversed in the PA+PEA group (*t* = 3.119, *p* < 0.01). This group did not present significant differences with controls (*t* = 0.462, *p* > 0.05) (Figure 4A). Similar results were found in the EPM test as regards time spent rearing. Birth condition and treatment were significant factors (*F* = 41.26, *p* < 0.0001; *F* = 10.69, *p* = 0.0024, respectively), and the interaction was also significant (*F* = 11.67, *p* = 0.0016). According to *post hoc* analysis of the simple effects, a significant reduction in time spent rearing was registered as a consequence of PA (*t* = 6.958, *p* < 0.001), and reversed after PEA treatment (*t* = 4.728, *p* < 0.001). PA+PEA group did not show significant differences with respect to controls (*t* = 2.126, *p* > 0.05) (Figure 5A). Both in OF and EPM test, treatment with PEA in CTL rats had no significant effect on time spent rearing in comparison to CTL rats injected with VHI (*t* = 0.4677, *p* > 0.05; *t* = 0.1042, *p* > 0.05, respectively) (Figures 4A, 5A, respectively).

Although birth condition was not significant for time spent in open arms in the EPM test (*F* = 3.203, *p* = 0.0813), other anxiety-related behaviors were encountered as a consequence of PA at P30. With regard to time spent grooming in the OF test, two-way ANOVA revealed birth condition and treatment were significant (*F* = 6.015, *p* = 0.0172; *F* = 16.64, *p* = 0.0001, respectively), but not the interaction (*F* = 3.405, *p* = 0.8767). *Post hoc* analysis indicated time spent grooming was significantly augmented as a result of PA (*t* = 2.88, *p* < 0.05), and reversed after PEA treatment (*t* = 4303, *p* < 0.001). PA+PEA group did not present significant differences with controls (*t* = 0.4561, *p* > 0.05) (Figure 4B). Time spent grooming was also altered in the EPM test as a consequence of PA, but PEA treatment did not exert a protective effect in this case. Two-way ANOVA indicated birth condition was significant (*F* = 14.15, *p* = 0.0007), in contraposition to treatment (*F* = 0.0001407, *p* = 0.9906) and the interaction (*F* = 0.02445, *p* = 0.8767). *Post hoc* analysis revealed time spent grooming was significantly increased in the PA group in comparison to the CTL group (*t* = 2.873, *p* < 0.05), but not reversed in the PA+PEA group (*t* = 0.09661, *p* > 0.05). This group presented significant differences with controls (*t* = 2.464, *p* < 0.05) (Figure 5B). In both OF and EPM tests, treatment with PEA in CTL rats had no significant effect on time spent grooming in comparison to CTL rats injected with VHI (*t* = 1.54, *p* > 0.05; *t* = 0.1267, *p* > 0.05, respectively) (Figures 4B, 5B, respectively). As for time spent HD (in the EPM test), two-way ANOVA indicated birth condition, treatment and the interaction were significant (*F* = 53.33, *p* < 0.0001; *F* = 33.4, *p* < 0.0001; *F* = 34.19, *p* < 0.0001, respectively). *Post-hoc* analysis of the simple effects revealed time spent HD was significantly augmented in the PA group (*t* = 9.781, *p* < 0.001), and reversed in the PA+PEA group (*t* = 7.99, *p* < 0.001). This group did not show significant differences with controls (*t* = 1.185, *p* > 0.05). No significant differences were found between CTL and CTL+PEA groups either (*t* = 0.04995, *p* > 0.05) (Figure 5C).

Finally, aversive associative memory might not be affected at P30 as a consequence of PA. Two-way ANOVA revealed birth condition was not significant with respect to T2/T1 ratio from the IA task (*F* = 0.854, *p* = 0.361). Treatment was not significant either (*F* = 0.0061, *p* = 0.931).

## Discussion

In the present work we investigated biochemical, morphological and behavioral alterations induced by the PA rat model at P30, since this time point corresponds to the age of NDDs onset in humans (Herrera-Marschitz et al., 2014, 2017). We also examined the effect that the neuroprotective treatment with PEA exerted at this sensitive time-window when deficits become evident (Meredith et al., 2012). Interestingly, our findings show vulnerability of CA1 hippocampal neurons to PA at P30. These neurons showed clear signs of degeneration, including NeuN fragmentation of the nucleus, cytoplasmic staining without nuclear staining, and nuclear pyknosis. Neuronal cytoskeleton was affected as it can be inferred from an excessive accumulation of pNF-H/M and a significant reduction in MAP-2 marker. The functional correlate was associated with changes in prototypical behaviors, which indicate vertical exploration impairments and altered anxiety levels. PEA treatment (10 mg/kg) within the first hour of life could attenuate these alterations at P30, which precede well-known long-term impairments induced in the PA rat model (Capani et al., 2009; Saraceno et al., 2010, 2012; Galeano et al., 2011, 2015; Muñiz et al., 2014).

In previous works from our laboratory, the effect of PA was evaluated using both cesarean and vaginal fostered controls. Blanco et al. (2015) reported no differences in most of the behavioral, cellular and molecular parameters assessed using cesarean and vaginal controls. The effect of PA at P30 was also assessed using vaginal fostered control pups and major differences were found between both groups (Saraceno et al., 2016). Therefore, considering these results and the fact that we subject animals to a severe PA (19 min), we consider that differences between groups can be attributed mainly to the effect of PA insult. Additionally, in the present work, we mixed asphyctic rats with control rats in order to provide the same mother and postnatal environment, and asphyctic rats were well accepted by these surrogate mothers (see Materials and Methods section).

### Neuronal cytoskeleton modifications induced by PA at P30

Previous studies have shown that neurofilaments change their degree of phosphorylation after cerebral hypoxia-ischemia (Mink and Johnston, 2000) and serve as clinical biomarkers of hypoxic-ischemic encephalopathy (Douglas-Escobar et al., 2010). Dendritic alterations are also a common finding under glucose/oxygen deprivation (Park et al., 2008) and hypoxic-ischemic injury (Zhu et al., 2003; Takita et al., 2004). A 40% decrease of MAP-2-positive cells/mm^3^ was observed in organotypic hippocampal cultures from asphyxia-exposed animals (Morales et al., 2007). Since MAP-2 is a cytoskeletal protein, its expression depends on ATP concentration, which is reduced as a result of hypoxia (Ashworth et al., 2003). Calpain-induced proteolysis of MAP-2 may be an initial response to a hypoxic insult (Johnson and Jope, 1992). In agreement with this background, our results reveal a significant increase of pNF-H/M and a reduction of MAP-2 as a result of PA at P30. These findings suggest pNF-H/M and MAP-2 constitute promising biomarkers of short-term PA-induced damage in CA1, which can be considered as an initial degenerative process of neurons, as it can be inferred from our electron microscopy analyses. In addition, dendritic alterations (Matesic and Lin, 1994) and aberrant accumulation of pNF-H/M (Dale and Garcia, 2012) are well-known hallmarks of neurodegeneration in several diseases.

### Functional correlate of neuronal cytoskeletal alterations at P30

Recent evidence suggests hyperphosphorylation of neurofilaments (Chen et al., 2017) and decreased MAP-2 levels (Soares et al., 2016) in the hippocampus are correlated with cognitive and anxiety dysfunctions in murine models of neurodegeneration and global cerebral ischemia, respectively. In the present study, PA-induced hippocampal accumulation of pNF-H/M and reduction of MAP-2 at P30 exhibited a similar behavioral correlate. PA induced a significant decrement in time spent rearing, a hippocampal-dependent behavior which accounts for vertical exploration in response to novelty (Lever et al., 2006). We hypothesize exploration could have been modulated by excessive grooming displayed by PA rats. Long time spent grooming has been associated with increased anxiety levels (Kalueff and Tuohimaa, 2005), which tend to interfere on free exploration (Lever et al.). In addition, the significant augmentation encountered in time spent HD suggests PA induced an increase in risk assessment behaviors (RAB), which might also alter exploratory activity.

Alterations in rearing, grooming and HD at P30 might precede long-term impairments in locomotion and horizontal exploration (Galeano et al., 2011). In fact, these PA-induced modifications in prototypical behaviors at P30 could not be attributed to impairments in locomotion or general activity, since no differences were observed in number of line crossings, time spent in closed arms, and total distance traveled in OF and EPM tests. Unlike the OF and the EPM tests, the IA task might not be sensitive to early changes induced by PA. Aversive associative memory was not impaired at P30, reproducing previous findings from our laboratory (Saraceno et al., 2016).

### Glial response to PA at P30

Unlike pNF-H/M and MAP-2, GFAP might not be an appropriate marker at P30. No significant differences were observed in the number of GFAP astrocytes or in GFAP expression 1 month after PA. Similar results were reported in previous studies from our laboratory (Saraceno et al., 2016), where we suggested PA-induced modifications in astrocyte population might be progressive and sustained long after PA. Astrogliosis is generally reported in a tertiary phase post-insult, occurring months after PA (Douglas-Escobar and Weiss, 2015). In this sense, we have previously observed a markedly significant increase in the number of GFAP immunoreactive astrocytes at P120 (Saraceno et al., 2010). Consistent with our current and previous findings, recent clinical evidence suggests GFAP is not an early marker of injury in PA (Looney et al., 2015).

### Neuroprotective effect of PEA treatment

PEA treatment (10 mg/kg) displayed protective effects by attenuating PA-induced degeneration and cytoskeletal alterations of CA1 neurons. As for microglia, even when PEA is produced by microglial cells and has a great effect on their modulation (Walter et al., 2003; Guida et al., 2017b), rodents subjected to hypoxic-ischemic injury present low microglial activity at P30. Therefore, the pharmacological inhibition of microglia at P30 could worsen the outcome after PA (Ferrazzano et al., 2013). PEA could also reverse exploration deficits and modulate anxiety levels at P30. *In vitro* studies in organotypic hippocampal slices have shown neuroprotective effects of PEA via PPAR-α (Koch et al., 2011; Scuderi et al., 2012). This receptor and its endogenous ligand, PEA, are involved in several cognitive and emotional processes (Fidaleo et al., 2014).

Although PEA endogenous content tends to increase around P30 in physiological conditions (Lee et al., 2013), a dysregulation of NAEs signaling system is induced by PA at this time point (Blanco et al., 2015; Holubiec et al., 2017). One month after PA, the expression of enzymes responsible for synthesis (diacylglycerol lipase-α–DAGL-α, and *N*-acyl-phosphatidylethanolamine-NAPE-hydrolyzing phospholipase D—NAPE-PLD) and degradation (fatty acid amide hydrolase—FAAH) of PEA, and its receptor PPAR-α, is decreased in hippocampal CA1 (Blanco et al.). Therefore, the neuroprotective effects of PEA on PA we observed in CA1 at this time point might be attributable to a counter regulation of this signaling system through the exogenous administration of PEA. In addition, hippocampal sensitivity to NAEs signaling might also contribute to explain the neuroprotective action of PEA treatment in this brain area and its dependent behaviors. Recent evidence has supported the deleterious effects of NAEs dysregulationon control of neurogenesis, neuronal death and gliosis, in a regional-dependent manner, presenting the hippocampus specific vulnerability (Rivera et al., 2015).

## Conclusion

PEA treatment (10 mg/kg) within the first hour of life could attenuate PA-induced alterations in CA1 neurons at P30, including nuclear pyknosis, aberrant accumulation of pNF-H/M in axons, and reductions in MAP-2 dendritic protein. These effects were associated with significant improvements in exploratory activity and a regulation of anxiety levels. Therefore, PEA represents a putative neuroprotective agent for PA-induced hippocampal alterations at P30. Future studies should focus on the protective effect of PEA treatment on PA at earlier and later developmental stages.

## Author contributions

MIH: Writing and Investigation. LDU, NT-U, CFK, and JPL: Investigation. FC: Supervision.

### Conflict of interest statement

The authors declare that the research was conducted in the absence of any commercial or financial relationships that could be construed as a potential conflict of interest.
